# Supplementary material for: Conditional chemoconnectomics (cCCTomics) as a strategy for efficient and conditional targeting of chemical transmission
Source: eLife. 2024 Apr 30;12:RP91927. doi: 10.7554/eLife.91927 (PMC11060718; doi:10.7554/eLife.91927)
Supplement: Figure 3—source data 3. [file elife-91927-fig3-data3.zip › Figure 3 source data3 part 1.zip!/Figure 3-Source data 3-part1/1071f2-1-CRISPResso_Report_ztwktvfp.zip!/CRISPResso_on_1071f2_2/9.Alleles_frequency_table_around_sgRNA_gcagaaggatttgtaaatgg.pdf]

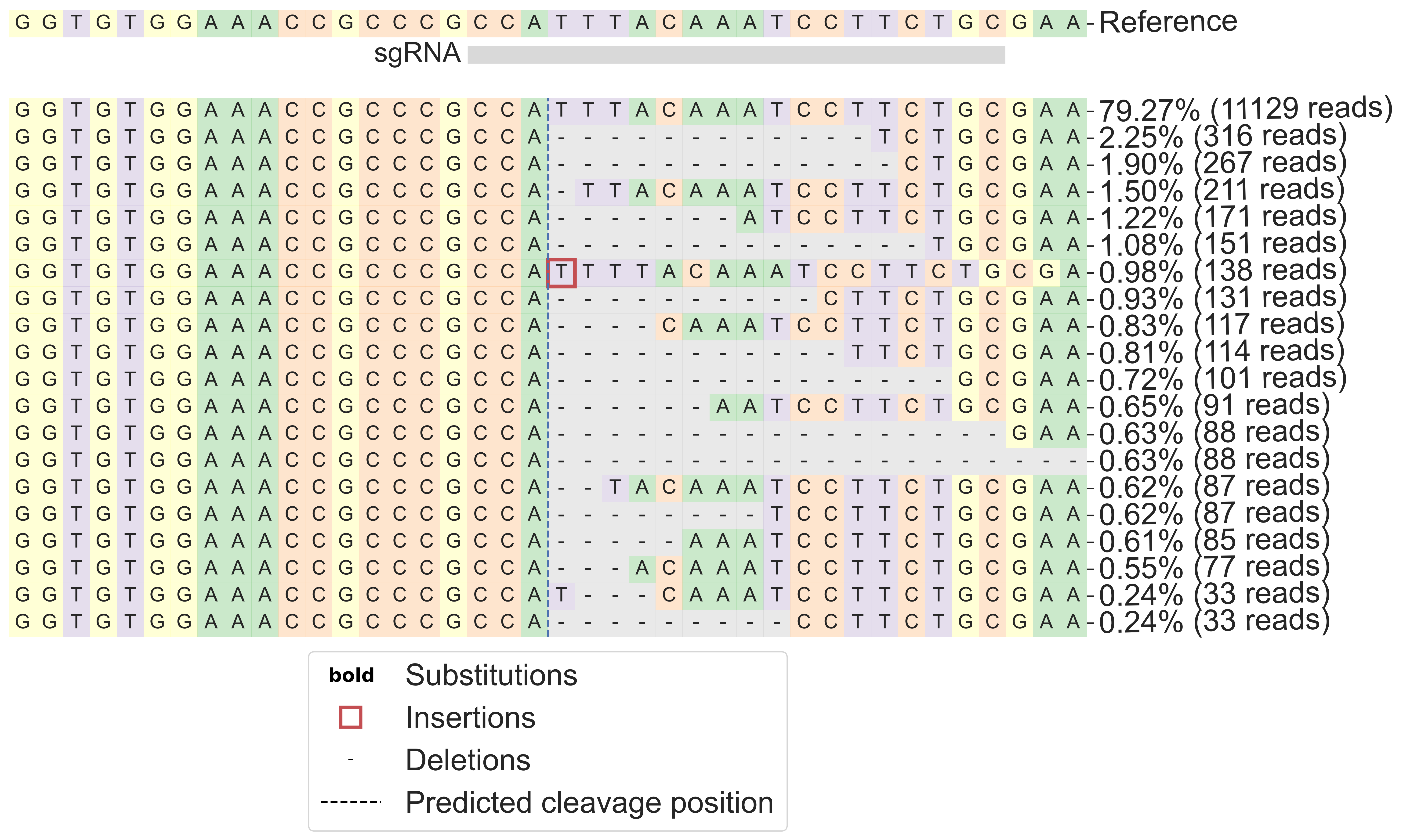
GGTGTGGAAACCGCCCGCCATTTACAAATCCTTCTGCGAA Reference
sgRNA
GGTGTGGAAACCGCCCGCCATTTACAAATCCTTCTGCGAA 79.27% (11129 reads)
GGTGTGGAAACCGCCCGCCA------------TCTGCGAA 2.25% (316 reads)
GGTGTGGAAACCGCCCGCCA-------------CTGCGAA 1.90% (267 reads)
GGTGTGGAAACCGCCCGCCA-TTACAAATCCTTCTGCGAA 1.50% (211 reads)
GGTGTGGAAACCGCCCGCCA-------ATCCTTCTGCGAA 1.22% (171 reads)
GGTGTGGAAACCGCCCGCCA--------------TGCGAA 1.08% (151 reads)
GGTGTGGAAACCGCCCGCCATTTTACAAATCCTTCTGCGA 0.98% (138 reads)
GGTGTGGAAACCGCCCGCCA----------CTTCTGCGAA 0.93% (131 reads)
GGTGTGGAAACCGCCCGCCA----CAAATCCTTCTGCGAA 0.83% (117 reads)
GGTGTGGAAACCGCCCGCCA-----------TTCTGCGAA 0.81% (114 reads)
GGTGTGGAAACCGCCCGCCA---------------GCGAA 0.72% (101 reads)
GGTGTGGAAACCGCCCGCCA------AATCCTTCTGCGAA 0.65% (91 reads)
GGTGTGGAAACCGCCCGCCA-----------------GAA 0.63% (88 reads)
GGTGTGGAAACCGCCCGCCA-------------------- 0.63% (88 reads)
GGTGTGGAAACCGCCCGCCA--TACAAATCCTTCTGCGAA 0.62% (87 reads)
GGTGTGGAAACCGCCCGCCA--------TCCTTCTGCGAA 0.62% (87 reads)
GGTGTGGAAACCGCCCGCCA-----AAATCCTTCTGCGAA 0.61% (85 reads)
GGTGTGGAAACCGCCCGCCA---ACAAATCCTTCTGCGAA 0.55% (77 reads)
GGTGTGGAAACCGCCCGCCAT---CAAATCCTTCTGCGAA 0.24% (33 reads)
GGTGTGGAAACCGCCCGCCA---------CCTTCTGCGAA 0.24% (33 reads)
bold Substitutions
Insertions
- Deletions
------ Predicted cleavage position
